# Stage-Specific Expression and Subcellular Localization of Calcineurin in Infective Forms of *Leishmania amazonensis*

**DOI:** 10.3390/pathogens14111139

**Published:** 2025-11-10

**Authors:** Deborah Brandt-Almeida, Ismael Pretto Sauter, Mario Costa Cruz, Cristian Cortez, Patricio Reyes Orrego, Mauro Cortez

**Affiliations:** 1 Department of Parasitology, Institute of Biomedical Sciences, University of São Paulo, São Paulo 05508000, SP, Brazil; deborah.brandt@usp.br (D.B.-A.); ipsauter@gmail.com (I.P.S.); 2 Core Facilities to Support Research, Institute of Biomedical Sciences, University of São Paulo, São Paulo 05508000, SP, Brazil; mccruz@usp.br; 3 Escuela de Tecnología Médica, Facultad de Ciencias, Universidad de San Sebastián, Santiago 8420000, Chile; cristian.cortez@uss.cl; 4 Departamento Biomédico, Facultad de Ciencias de la Salud, Universidad de Antofagasta, Antofagasta 1270300, Chile; patricio.orrego@uantof.cl; 5 Escuela de Tecnología Médica, Facultad de Ciencias, Pontificia Universidad Católica de Valparaíso, Valparaíso 2373223, Chile

**Keywords:** Amastigotes, Calcineurin, CaNA, CaNB, *Leishmania amazonensis*, promastigotes

## Abstract

Calcineurin (CaN), a Ca^2+^-dependent phosphatase, plays key roles in eukaryotic cell signaling. We investigated whether *Leishmania amazonensis*’ two infective forms—promastigotes and amastigotes—exhibit differences in CaN expression, localization, and functional impact, using two canonical inhibitors (cyclosporin A, CsA; tracolimus, FK506). At high 40 µM CsA, promastigotes showed reduced viability, whereas amastigotes remained resistant. FK506 had no effect on either form. At a sub-lethal 25 µM CsA, parasite proliferation remained unaffected. In parasite–macrophage co-incubation assays, phosphorylation patterns differed: amastigotes—but not promastigotes—showed increased serine/threonine phosphorylation upon CaN inhibition. Western blotting and in silico data revealed higher CaN catalytic (CaNA2) and regulatory (CaNB) subunit expression in amastigotes than promastigotes. Immunofluorescence localized CaNA prominently in both cytoplasm and nucleus of promastigotes, but predominantly cytoplasmic in amastigotes; CaNB was largely cytoplasmic in both. In silico localization predictions suggested strong membrane associations for CaNA in *Leishmania*, contrasting with mammalian models. Subcellular fractionation confirmed CaNA enrichment in membrane fractions, with CaNB in cytoplasmic and nuclear fractions. Collectively, these findings reveal form-specific differences in expression, subcellular distribution, and inhibitor responses of CaN in *L. amazonensis*, highlighting its potential as a stage-specific therapeutic target in leishmaniasis.

## 1. Introduction

Leishmaniasis remains one of the most prevalent neglected tropical diseases, second only to malaria in parasitic mortality rates, with up to one million new cutaneous cases and tens of thousands of visceral cases annually [1]. From the different manifestations, *Leishmania amazonensis* is among the most common species associated with the cutaneous form, including localized cutaneous leishmaniasis and diffuse cutaneous leishmaniasis (DCL), a more severe form of the disease [2]. The disease is complex and caused by protozoan parasites of the genus *Leishmania*, which are transmitted to humans and other mammals through the bite of infected female sandflies [3]. *Leishmania* parasites undergo a digenetic life cycle, alternating between the flagellated, extracellular promastigote form in the sandfly vector and the non-flagellated, intracellular amastigote, dwelling within vertebrate macrophages [4,5]. These two forms differ morphologically, metabolically, and in their interactions with host cells [6], driven by major shifts in pH, temperature, and nutrient availability [5,7].

High-throughput transcriptomic and proteomic analyses have revealed significant differences between promastigote and amastigote stages [7,8], including variations in metabolic and translation machinery proteins, virulence factors, and stress-response signals [4,9,10]. However, the functional role of phosphorylation-based regulation—specifically phosphatases—in stage-specific biology remains less explored. Protein phosphatases are known to contribute to *Leishmania* survival, adaptation, and virulence by modulating host signaling and within-parasite pathways [1,9]. Interestingly, phosphatases secreted by promastigotes or amastigotes can act synergistically with host cell phosphatases or act independently, modulating the response of infected cells [1].

Calcineurin (CaN), a calcium- and calmodulin-dependent Ser/Thr phosphatase, orchestrates critical processes including stress response and morphogenesis across eukaryotes [11,12]. CaN is a heterodimer formed by the catalytic subunit (CaNA) with a mass between 57 and 59 kDa (mammals) or between 57 and 71 kDa (lower eukaryotes) depending on the isoform, and a regulatory subunit (CaNB) of 19 to 20 kDa [13,14]. CaNA is highly conserved in eukaryotes; the *CaNA* genes encode a polypeptide consisting of a catalytic domain homologous to other protein phosphatases and three regulatory domains at the C-terminus. These last three domains have been identified as CaNB ligands [15,16], the CaM-binding domain [17,18], an autoinhibitory sequence (AIS) that blocks a substrate recognition site essential for LxVP motifs [19], and the autoinhibitory domain AID [20,21]. CaNB is highly conserved and contains four Ca^2+^-binding “EF-hand” motifs [22]. Thus, the CaN activation mechanism is established when intracellular Ca^2+^ levels increase, inducing conformational changes in CaNB, which facilitates CaM binding, displacing AIS and AID, and leaving the active site of CaNA available for interaction with the substrate [19,21].

In eukaryotic microbial pathogens, CaN controls essential virulence pathways, which are conserved in eukaryotic microbial organisms [23], including adaptation to host temperature, morphogenesis to enable invasive hyphae growth, drug tolerance and resistance, cell wall integrity, and sexual development. This is why the CaN signaling pathway is an attractive target for the development of drugs against eukaryotic pathogens [24]. It is important to mention that components such as CaM and CaN show differences among microorganisms, such as some pathogenic fungi and some pathogenic protists [24]. In pathogenic yeast-like fungi, the most studied target is Crz1 [25], which, when dephosphorylated, translocates to the nucleus and activates the expression of various genes, including those involved in chitin synthesis, *CHS7*, and membrane transport *PMC1* [26], as well as *CMK2*, a yeast homolog of *CaMKII* [27]. However, targets other than *Crz1* have also been described in the yeasts *Saccharomyces cerevisiae*, *Cryptococcus neoformans*, and *Candida albicans*, as well as in the filamentous fungus *Aspergillus fumigatus* [24]. Clarifying where and when CaN acts is essential for mapping CaN and its substrates to specific Ca^2+^ signaling microdomains throughout the cell, and disruption of these local interactions provides exquisite insight into the discrete functions of each localized signaling event [28]. To date, CaN in *Leishmania* has been implicated in environmental adaptation within the mammalian host, yet differential regulation of CaN across life stages remains unexplored [29] and represents a potential biomarker of resistance to antileishmanial drugs [30].

The differential domain structure of the CaNA and CaNB subunits, their subcellular localization, their potential post-translational modifications such as proteolytic fragmentation, and their possible role in the extracellular environment, among other mechanisms, underscore the importance of understanding the biological role of CaN in the biology of intracellular pathogenic protozoa such as *Leishmania* [12,31]. The lack of transcription factors in the genomes of parasitic protozoa suggests that the CaN pathway may operate in ways other than the transcription factor-mediated regulatory mechanisms that control the virulence of parasitic protists [32], suggesting that the CaN signaling pathway could function in ways other than the mechanisms described, thus conditioning their virulence in parasites such as *Leishmania*.

We hypothesized that CaN differs in expression, subcellular localization, and functional relevance between promastigote and amastigote forms of *L. amazonensis*. Here, we present experimental evidence demonstrating that amastigotes express higher CaN levels and display distinct subcellular distributions compared to promastigotes, with differential sensitivity to CsA. Together, these findings illuminate stage-specific regulation of CaN and suggest novel therapeutic avenues targeting CaN in leishmaniasis.

## 2. Materials and Methods

### 2.1. Bone Marrow-Derived Macrophages Isolation

Bone marrow-derived macrophages (BMM) from C57BL/6 mice were generated after 7 days of bone marrow differentiation as previously described [33]. All experiments were carried out in accordance with internationally recognized ARRIVE guidelines and in full agreement with institutional and local regulations [Brazilian Federal Law #11794, Decree #6899 and Normative Resolutions published by the National Council for the Control of Animal Experimentation (CONCEA)] and approved by the Institutional Animal Care and Use Committee (IACUC) under protocol #7433061223 at the Institute of Biomedical Sciences, University of São Paulo, Brazil.

### 2.2. Leishmania amazonensis Parasites

*Leishmania amazonensis* (IFLA/BR/67/PH8) parasites were obtained from lesions in C57BL/6 mice and then propagated as promastigotes in M199 medium (Vitrocell, Campinas, SP, Brazil) supplemented with 40 mM HEPES, 2.5 µg/mL hemin, 10 mM adenine, 2 mM L-glutamine, 2 µg/mL D-biotin, 100 U/mL penicillin, 100 µg/mL streptomycin, and 20% (*v*/*v*) iFBS, pH 7.2, at 26 °C in a bio-oxygen demand incubator, as described [34,35]. Subcultures were made weekly at an initial density of 5 × 10^5^ promastigotes/mL up to six passages. Parasite cultures enriched in metacyclic promastigotes (approximately 25% from the parasite culture were obtained from a stationary phase promastigote maintained for a minimum of 6 to 7 days. To generate axenic amastigotes, stationary phase promastigote cultures were incubated at 2 × 10^6^/mL in M199 media containing 0.25% glucose, 0.5% trypticase, 40 mM sodium succinate (at pH 4.5), 20% FBS, 5% penicillin/streptomycin at 32 °C for a minimum of 6 days, and then cultured axenically at 32 °C. Parasites were washed 3 times in phosphate-buffered saline (PBS) before use in experiments.

### 2.3. CaN Inhibition and Cell Viability Assay

To inhibit parasite CaN, *Leishmania* infective forms were treated with different concentrations of Cyclosporin A (CsA, Sigma-Aldrich, St. Louis, MO, USA) or FK506 (Invivogen, San Diego, CA, USA) in their specific medium at 26 °C (promastigotes) or 32 °C (amastigotes), respectively, for 2 h. The parasites were washed 3 times with PBS, and the viability was accessed by MTT assay. Briefly, 5 × 10^6^ treated parasites per well were placed in a 96 well plate in fresh medium with 30 µL of 5 mg/mL MTT [3-(4,5-dimethyl-2-thiazolyl)-2,5-diphenyl-2H-tetrazolium bromide; Sigma-Aldrich, St. Louis, MO, USA] and kept at 26 or 32 °C for 4 h. The reaction was stopped by adding 30 µL 20% sodium dodecyl sulfate (SDS; Sigma-Aldrich, St. Louis, MO, USA) to each well and the optical density was determined in a plate reader (POLARstar Omega, BMG Labtech, Ortenberg, Germany) with a reference wavelength of 690 nm, and a test wavelength of 595 nm. Results were expressed as the mean percentage viability of treated compared to untreated parasites. To evaluate the effect of CaN inhibitors on the protein’s phosphorylation, *Leishmania* infective forms were incubated for 2 h at 26 or 32 °C, with 20 µM of CsA or FK506. After incubation, the parasites were washed 3 times with PBS, and proteins were extracted by using lysis buffer (Thermo Fisher Scientific, Waltham, MA, USA) supplemented with protease and phosphatase inhibitor cocktail (Thermo Fisher Scientific) supplemented with 1 mM sodium orthovanadate and processed by Western blot. To recognize some of the CaN substrate proteins, *Leishmania* infective forms were incubated in the presence of macrophage protein extracts. Briefly, amastigotes and promastigotes were washed 3 times with PBS and incubated with 50 µg of sonicated macrophage extracts in Tyrode buffer (140 mM NaCl, 5 mM KCl, 2.5 mM CaCl_2_, 10 mM HEPES, 2 mM MgCl_2_, pH 7.2, containing protease inhibitors cocktail). After 2 h, the parasites were treated with 20 µM of CaN inhibitors for another 2 h, lysed in the presence of phosphatase inhibitors cocktail, followed by Western blot.

### 2.4. Western Blot

Protein extracts of parasites (1 × 10^7^) or macrophages (20 µg) per well were separated under reducing conditions on a 12% sodium dodecyl sulfate (SDS) polyacrylamide gel and blotted onto nitrocellulose membranes with a transfer system (BioRad Laboratories, Hercules, CA, USA). The membranes were probed with a rabbit anti-mouse phosphoserine mAb, mouse anti-Tubulin monoclonal antibody (Cell Signaling Technology Inc., Danvers, MA, USA), rabbit anti-CaNA or anti-CaNB polyclonal antibodies [36], and rabbit anti-mouse actin mAb (Imuny-VBP Biotecnologia Ltd., São Paulo, SP, Brazil), followed by peroxidase-conjugated anti-rabbit IgG mAb (Imuny-VBP Biotecnologia Ltd., São Paulo, SP, Brazil),). Immunoblots were developed by using the Supersignal West Pico Chemiluminescent Substrate (Thermo Scientific) and detected with a ChemiDoc XRS Imaging System (BioRad Laboratories, Hercules, CA, USA) on the ImageLab (BioRad) 6.1 software.

### 2.5. Immunofluorescence

For CaN localization, *Leishmania* infective forms were fixed with 4% PFA in PBS, treated with 50 mM NH_4_Cl, and then treated with blocking and permeabilization solution for 30 min. After, the fixed parasites were incubated with anti-CaNA or anti-CaNB polyclonal antibody [36], followed by anti-mouse or anti-rabbit IgG antibody Alexa Fluor 488-conjugated (Thermo Fisher Scientific), respectively. Samples were incubated with 10 µg/mL DAPI (Sigma-Aldrich, St. Louis, MO, USA) to detect the nuclei/kinetoplast (artificially modified for red signal) of parasites. The images were randomly acquired in a fluorescence microscope (Leica DMI6000B/AF6000, Leica Microsystems, Wetzlar, Germany) coupled to a digital camera system (DFC365FX), moving through visual fields in parallel rows across each coverslip. An appropriate filter set was used depending on the sample fluorescence labeling. Confocal microscopy images were acquired in a confocal fluorescence microscope coupled to a digital camera system, and images were analyzed by Imaris software (https://imaris.oxinst.com/).

### 2.6. Prediction of the Subcellular Localization of CaN

The subcellular location of the CaN subunits in mouse (accession numbers NP_032939.1 and NP_077779.2 were retrieved from NCBI Reference Sequence) and *Leishmania amazonensis* (accession numbers LAMA_000326600 and LAMA_000415300 were retrieved from TriTrypDB) was carried on CELLO2GO: a web server for protein subcellular localization prediction with functional gene ontology annotation.

### 2.7. Statistical Analysis

Data were analyzed with Prism 6.0 (GraphPad Software, San Diego, CA, USA). Statistical significance was determined by One way ANOVA following Kruskal–Wallis test. Statistically significant differences were defined as * when *p*-values were < 0.05, ** *p* < 0.01, and *** *p* < 0.001. Results represent means ± standard deviation (SD) or standard error of the mean (SEM) as indicated. The number of independent experiments, technical and biological replicates are indicated in corresponding figure legends.

## 3. Results

### 3.1. CaN Inhibitors Differentially Affect Parasite Viability

**Figure 1:**
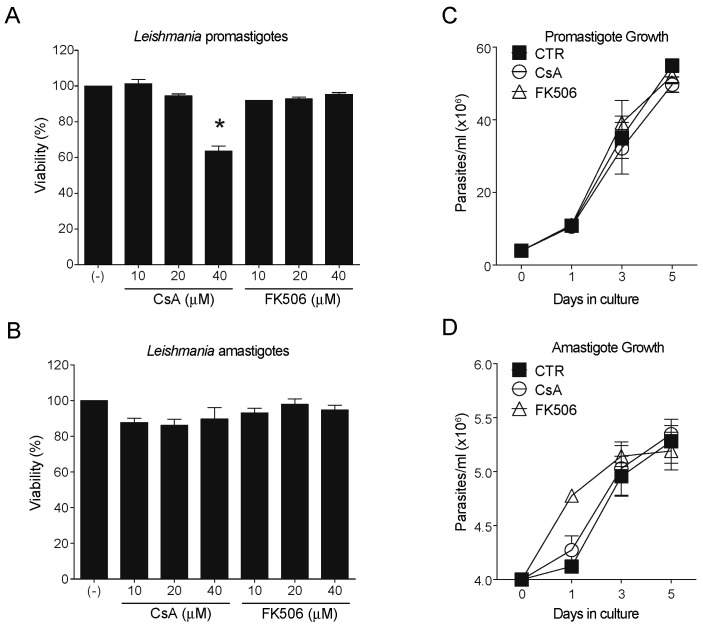
CaN inhibitors differentially affect parasite viability. (**A**) Promastigote parasites were treated with different concentrations of CsA (10, 20, and 40 μM) and FK506 (10, 20, and 40 μM) for 24 h. A significant reduction in viability was observed only at the 40 μM CsA concentration (>40% mortality), while the other treatments had no significant effect. (**B**) Amastigote parasites were subjected to the same treatment conditions and showed no significant changes in viability. To evaluate the impact of CsA and FK506 on proliferation, promastigotes (**C**) and amastigotes (**D**) were pretreated with 25 μM of the compounds, counted, and cultured for 5 days. Daily counting demonstrated that neither drug did not interfered with growth compared to the untreated control. Results represent means ± standard error of the mean (SEM) as indicated from three biological replicates. One-way ANOVA following Kruskal–Wallis test. * *p*-values < 0.05.

To evaluate the function of CaN in both stationary-phase promastigotes (enriched in metacyclic promastigotes, the infective form) and amastigotes, we first evaluated parasite viability by MTT with increasing concentrations (10–40 µM) of the classical CaN inhibitors, CsA and FK506. At 40 µM CsA, promastigotes exhibited approximately 40% mortality (Figure 1A), while amastigotes remained unaffected (Figure 1B). FK506 had no detectable toxicity on either form at the tested concentrations. Moreover, when both forms pretreated with CsA or FK506 were incubated in complete inhibitor-free medium, the parasites recovered the capacity to proliferate (Figure 1C,D). To investigate non-lethal effects, 25 µM CsA was used in subsequent assays, as this dose did not significantly alter parasite viability or proliferation compared with controls.

### 3.2. Phosphorylation Profile Altered by CaN Inhibition Only in Amastigotes

**Figure 2:**
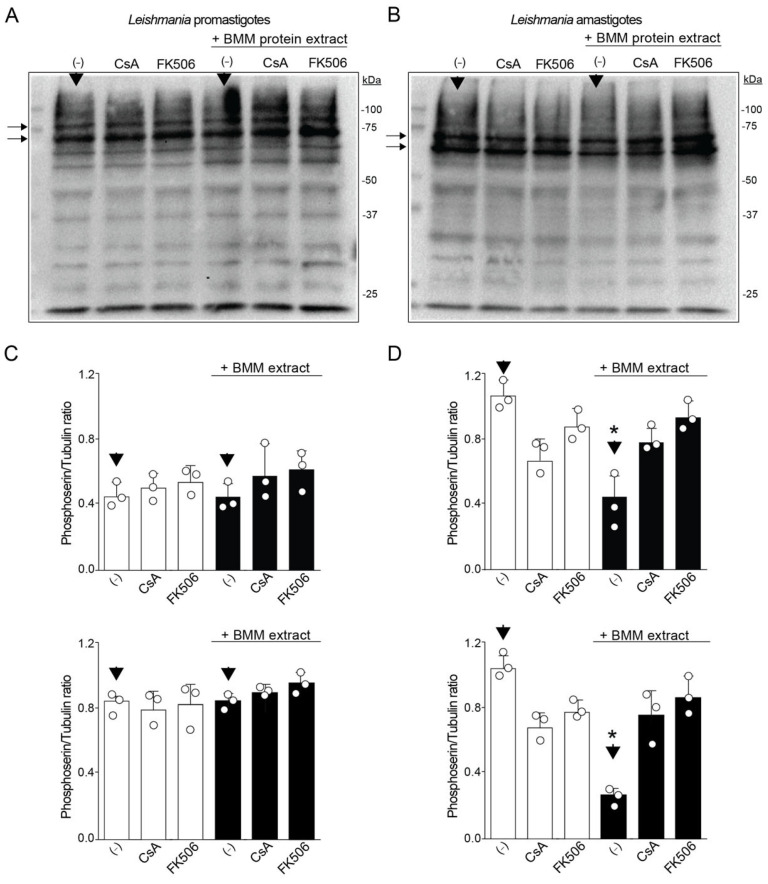
Phosphorylation profile altered by CaN inhibition only in amastigotes. Levels of serine-phosphorylated proteins from infective forms of *L. amazonensis* treated with CaN inhibitors. CsA and FK506-treated (**A**) promastigotes and (**B**) amastigotes were incubated with BMM protein extract (+) at different temperatures (26 and 32 °C) for 2 h. After incubation, the parasites were washed and lysed for Western blot by using anti-Phosphoserine/threonine antibodies. Arrows indicate different phosphorylated proteins analyzed. The molecular weight marker (kDa) is displayed on the right side of both images. Densitometry analysis of the two proteins indicated in A and B by arrows, using tubulin as protein load (ratio Phosphorylated protein/tubulin) from (**C**) promastigote and (**D**) amastigotes samples. The graph represents the densitometry of triplicates. Results represent means ± standard error of the mean (SEM) as indicated from three biological replicates. One way ANOVA following Kruskal–Wallis test. * *p*-values < 0.05.

Several phosphoserine-protein targets could be dephosphorylated by the phosphatase activity of CaN [37]. To recognize some of the substrate proteins, *Leishmania* infective forms were treated with CaN inhibitors in the presence of macrophage protein extracts. Since several proteins were observed in each parasite protein extract, we were interested in identifying which protein diminished the level of phosphorylation compared to the untreated samples (-) with or without macrophage protein extracts (black arrows), analyzed by densitometry (Figure 2A,B). Thus, when macrophage protein extracts were incubated with promastigotes or amastigotes in the presence or absence of CaN inhibitors, the serine phosphorylation profiles diverged: promastigotes showed two prominent bands (~75 kDa and higher, lateral black arrows in A), unchanged by CsA or FK506. On the other hand, amastigotes displayed two main bands (~75 kDa and lower, lateral black arrows in B) whose intensity decreased in the presence of macrophage extract but increased upon CaN inhibition (20 to 40% for CsA and 48 to 50% for FK506), demonstrating CaN activity on the phosphorylation profile (Figure 2C,D). Together, these results provide evidence for the differences in CaN activities depending on the infective form of *L. amazonensis*.

### 3.3. Differential Expression of CaN Subunits in Leishmania amazonensis

**Figure 3:**
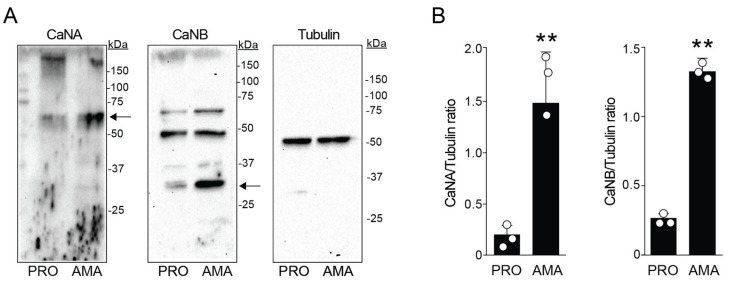
Differential expression of CaN subunits in *Leishmania amazonensis*. (**A**) Western blotting analysis of protein extracts from promastigote (PRO) and amastigote (AMA) forms using antibodies specific for the catalytic (CaNA) and regulatory (CaNB) subunits of CaN. Tubulin was used as a loading control. Increased detection of both subunits is observed in amastigotes compared to promastigotes. (**B**) Densitometric analysis of blots normalized to tubulin (CaN subunit/tubulin ratio). The results demonstrate that both CaNA and CaNB are significantly more highly expressed in amastigote forms compared to promastigote forms. Results represent means ± standard error of the mean (SEM) as indicated from three biological replicates. One way ANOVA following Kruskal–Wallis test. ** *p*-values < 0.001.

To determine the expression of CaN components in both infective forms of *Leishmania* by Western blots, polyclonal antibodies were used against CaNA/CaNB generated from *Trypanosoma cruzi* recombinant proteins [36], due to the high similarity with *Leishmania* CaN. In *L. amazonensis*, CaNA appeared as a ~60 kDa band, while CaNB showed three bands (~60, 45, and 30 kDa) (Figure 3A). Because it was demonstrated that CaNA and CaNB in *T. cruzi* have sizes of 43 and 19 kDa, respectively [38], we analyzed the closest in size to these subunits in our study. Densitometry analysis of the ratio from each CaN with tubulin (used as a control load) confirmed that both CaNA and CaNB were more expressed in amastigotes than promastigotes (Figure 3B).

When in silico analysis were performed of transcriptomic data from *Leishmania* infection, using databases deposited by different groups [39,40,41], we verified that intracellular amastigotes infecting macrophages presented an increased level of CaNA (analyzing CaNA2), and CaNB over infection time, when compared to the promastigotes (Supplementary Figure S1A,B). We also analyzed in these transcriptomic data a different isoform named CaNA1_var, but the levels were similar in both infective forms of *Leishmania* (Supplementary Figure S1C).

### 3.4. Subcellular Localization of CaNA and CaNB Differs Between the Infective Forms

**Figure 4:**
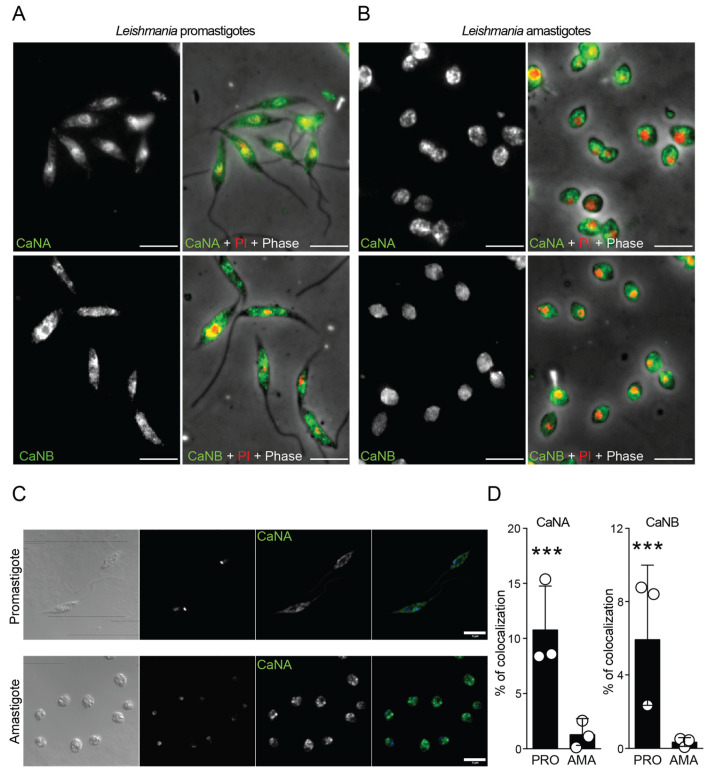
Subcellular localization of CaNA and CaNB differs between the infective forms. (**A**) Promastigotes and (**B**) amastigotes were fixed in coverslip-polylysine and processed by using immunofluorescence (IF). The parasites were sequentially stained with specific antibodies against CaNA and CaNB and anti-IgG rabbit Alexa 488 (green). The nuclei and kinetoplast were stained with propidium iodide (red), and the phase contrast channel was merged to recognize the form of the parasites. Bar = 5 μm. (**C**) New samples were prepared using the same antibodies above (**A**,**B**), but the nuclei/kinetoplast were stained with DAPI for further percentage of colocalization analysis. (**D**)The graphs represent the percentage (%) of colocalization CaN subunit/nuclei, analyzed for the two infective forms (PRO and AMA), measuring 50 events, counted for biological triplicates. Results represent means ± standard error of the mean (SEM) as indicated from three biological replicates. One way ANOVA following Kruskal–Wallis test. *** *p*-values < 0.0001.

Concerning CaN localization, and using immunofluorescence microscopy, we demonstrated that CaNA (stained in green) from promastigotes co-localized with propidium iodide (PI), which stains the nuclei of the parasites in red. However, CaNB was found mainly in the cytoplasmic region (Figure 4A). On the other hand, CaNA and CaNB are localized mainly in the cytoplasmic region in amastigotes (Figure 4B). To properly analyze and quantify the percentage of colocalization nuclei/CaN, we obtained confocal images from both promastigotes and amastigotes stained with the different CaN subunits (Figure 4C), which were analyzed by the Imaris software. Immunofluorescence microscopy showed that in promastigotes, CaNA localized to both cytoplasm and nucleus, colocalizing with DAPI. CaNB was mainly cytoplasmic. In contrast, both subunits were predominantly cytoplasmic in amastigotes, with little nuclear colocalization (Figure 4D).

These data revealed a remarkable difference between *L. amazonensis* promastigotes and amastigotes in the expression and localization of CaN, which strongly suggests a stage-specific function of this form in the infective process and opens up new avenues for further research.

**Figure 5:**
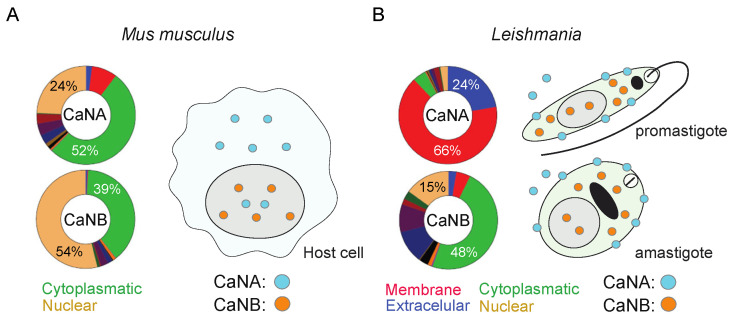
In Silico subcellular localization predictions of CaN in mouse, and *Leishmania*. (**A**) The predicted subcellular localization of CaNA and CaNB in *Mus musculus* (mouse), and (**B**) *Leishmania* is shown, using the CELLO2GO web server. The subcellular localizations are represented in ring chart diagrams, evaluating the significant terms in the form of their percentage contribution. In each one, the value with the highest probability is shown.

Finally, comparative prediction using mouse CaN subunits yielded cytoplasmic localization (green region) probabilities of 52% (CaNA) and 39% (CaNB), with nuclear predictions (light orange) of 24% and 54% respectively (Figure 5A). When the in silico analysis was performed in *Leishmania* CaN, strong differences were visualized. For *Leishmania* CaN subunits, CaNA was predicted to localize most strongly to membranes (66%) and extracellular space (24%), with negligible cytoplasmic or nuclear probability (<3%) (Figure 5B, upper diagram). CaNB predictions were more similar to the predictive localization of CaNA in the mouse, presenting a more cytoplasmic (48%) and nuclear (15%) prediction (Figure 5B, lower diagram).

## 4. Discussion

Promastigote and amastigote forms of *Leishmania* differ in several molecular and cellular processes related to survival and differentiation. Our results indicate that calcineurin (CaN) exhibits stage-specific differences in expression and subcellular localization.

Since its identification as a serine/threonine phosphatase [42], CaN has been linked to thermotolerance and infectivity in *Leishmania* [29]. However, its stage-specific role in parasite–host interactions remains insufficiently understood. Pharmacological inhibition using cyclosporin A (CsA) revealed stage-dependent effects on parasite viability: promastigotes were sensitive only at high CsA concentrations, whereas amastigotes responded at lower doses. These findings likely reflect the indirect action of CsA and FK506 through their binding to immunophilins—cyclophilin (Cyp) and FK506-binding protein (FKBP)—rather than direct inhibition of CaN [43].

In *L. major*, cyclophilin 19 (LmCyp19) binds CsA but does not interact with CaN [44], and CsA pre-treatment has been reported not to affect amastigote proliferation [45]. Our results are consistent with these observations, as CsA or FK506 treatment did not alter the phosphorylation pattern in promastigotes, even in the presence of macrophage extracts. These data suggest that immunophilins, rather than CaN itself, may have a more prominent role during early infection.

Conversely, in amastigotes, CaN inhibition altered serine phosphorylation patterns, suggesting that CaN contributes to stage-specific regulatory pathways involved in intracellular adaptation. The observed reduction in serine-phosphorylated proteins upon CaN inhibition indicates a possible direct role of CaN in amastigote signaling, with cyclophilins acting as secondary modulators [46,47].

Quantitative analyses revealed higher transcript and protein levels of CaNA2 and CaNB in amastigotes, consistent with enhanced CaN activity in this stage. Immunofluorescence and fractionation data showed that CaNA localizes partly to the nucleus in promastigotes but associates with membrane compartments in amastigotes, while CaNB remains cytoplasmic or nuclear. These patterns suggest distinct subcellular functions and potential adaptation to intracellular environments. Predictive modeling further supported membrane or extracellular localization of *Leishmania* CaNA, consistent with biochemical data and indicating divergence from mammalian CaN regulation.

Genomic analysis identified putative *Leishmania* CaN subunits—LaCaNA1, LaCaNA2, and LaCaNB—based on homology with *L. major* and *T. cruzi* orthologs [48,49,50]. In *T. cruzi*, CaNA1 and CaNA2 localize differently depending on parasite form [36,51]. Using antibodies against *T. cruzi* CaNA2 and CaNB [36,38], we observed that CaNA2 is nuclear and CaNB cytoplasmic in *L. amazonensis* promastigotes, while both are cytoplasmic in amastigotes. This may indicate a more stable CaNA2–CaNB complex in amastigotes, potentially supporting intracellular survival through calcium-dependent regulation.

Transcriptomic data from infected macrophages showed increased expression of LaCaNA2 and LaCaNB at 72 h post-infection, while LaCaNA1 expression decreased [39]. These expression trends are consistent with a possible role of the CaNA2–CaNB complex in intracellular infection. Although this is the first study to describe a stage-specific association between these subunits in *Leishmania*, further work is required to define their precise contribution to parasite virulence.

The upregulation of CaNA2 and CaNB in amastigotes, coupled with their membrane and cytoplasmic enrichment, suggests that CaN is actively involved in the signaling processes that govern differentiation and intracellular persistence. These results are consistent with findings in other trypanosomatids, where CaN activity has been associated with stress tolerance, cell cycle regulation, and host cell invasion [36,38,44,51]. In *L. amazonensis*, the enhanced CaN expression may represent an adaptive mechanism to counter oxidative or nitrosative stress encountered within macrophage phagolysosomes. Given that CaN integrates calcium and calmodulin signaling, its activation could serve as a key regulatory node linking environmental sensing to gene expression and metabolic adaptation during infection [52,53,54].

Finally, emerging evidence has associated CaN genes with anti-leishmanial drug resistance, particularly in *L. infantum* [33]. This raises the possibility that CaN not only supports parasite survival during infection but may also contribute to resistance mechanisms under drug pressure. However, the precise relationship between CaN transcript abundance, enzymatic activity, and resistance phenotypes remains to be clarified. It will be crucial to integrate transcriptomic, proteomic, and phosphoproteomic approaches to better define the molecular networks governed by CaN and its potential as a target for chemotherapeutic intervention.

## 5. Conclusions

This study provides evidence for stage-specific expression and localization of CaN in *Leishmania amazonensis*, with the CaNA2–CaNB complex potentially contributing to intracellular adaptation and survival. While these findings suggest a functional role for CaN during infection, further research is required to determine its precise mechanisms and relevance to pathogenesis and treatment.

## Data Availability

The original contributions presented in the study are included in the article. Further inquiries can be directed to the corresponding author.

## Supplementary Materials

The following supporting information can be downloaded at: https://www.mdpi.com/article/10.3390/pathogens14111139/s1, Figure S1: *Leishmania amazonensis*—MHOM/BR/71973/M2269 (Reference Strain) obtained from TriTrypDB.org. References cited in Supplementary Materials [39,40,41]. File S1: Original Western blot images.

## Abbreviations

The following abbreviations are used in this manuscript:

CaN	Calcineurin
PBS	phosphate-buffered saline
