# Supplementary material for: Stage-Specific Expression and Subcellular Localization of Calcineurin in Infective Forms of Leishmania amazonensis
Source: Pathogens. 2025 Nov 10;14(11):1139. doi: 10.3390/pathogens14111139 (PMC12655812; doi:10.3390/pathogens14111139)
Supplement: Supplementary file 1 [file pathogens-14-01139-s001.zip › pathogens-3900273-supplementary/pathogens-3900273-Figure S1.pdf]

Supplementary Figure S1.

***Leishmania amazonensis* - MHOM/BR/71973/M2269 (Reference Strain) obtained from TriTrypDB.org**

Based on comparative RNA-seq analysis of gene expression changes that occur when macrophages are infected with *Leishmania* spp.

A. *La*CaNA2 (407)

- LAMA_000326600 Calcineurin-like phosphoesterase, putative

MSQPNMKCSAVGGRNLNGTPVERNTLLSDDKLSLESIMLQFLCGEQLRLEYAIEIVQQAALVLRTEPNALSINDTVVVVGDVQGQYYDLVKILAACGSLSTTTYL
FLGNYIGNGGFNLECILFLLAAKVAHPQSIFLIRGSNESKFMADVLELGKECQLKYSSTLLPQILSAFNCLPLAAIIRKKFFCVHSGLSPDVSHVDDIGLIHRFRHIPT
RGAMCDMVWSEPDWDTNNQLYNNVEEPSGETYVPRLGSFETRPLFITNKQRGLSYVFNFACAKRFVSANNLLCIIRAHEVHELGFKLYRPHPNHLFPCIISLFSAP
NYCSSFGNKGAVLVVSKETICFKQFEPSPHPCVLQGRNAFSWSLPFLESNLMSIFLTLLSGSDFDPTVADRSEKGSSNGEGGATPLI

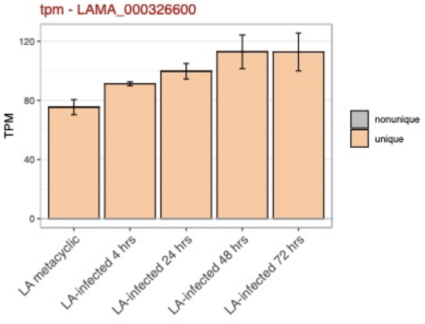

B. *La*CaNB (175)

- >LAMA_000415300

MNEVPLTAEELQYIRESTALTDAQVQRLYKSFSRLNKDKSGKITRAEFNSIPALASNPLVDRVLAVMDTDGDSTVDFGDFVRALAVLSSATSKEDKLRFTFKMYD
IDGDGRISNRDLFQMLSIMVGVNLSQMQLQQIVDKTFIEADVDRDGYITFEEFEALAVNSDFGDRLNLHF

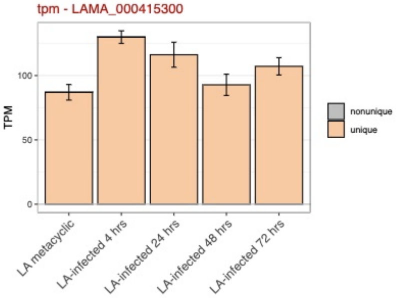

C. *La*CaNA1_var (503)

- LAMA_000526700 Calcineurin-like phosphoesterase, putative

MLLTDRGNPSVPPPNWEPLSSSALFDESGKVRPDVVREHLRKEGLLQEADALTIITQCALIWKDEPNVLRLDGSVAIAGDIHGQFFDLLNLLSIGGDPSQQKYIFL
GDYVDRGCFGMEVILLLMCYKICYPKTMIMLRGNHESRHLTAYFNFKREVLYKYSIAVYNAMMSAFDCLPLACILNNRFLCVHGGLSPELKRISDIGAIHRFREPP
SSGPMCDLLWADPLDEKEEDPAAAPLFVPNTTRGCSYVYSNAATCNFLKENDLITIIRGHEAQNEGYHLYKKTRKGFPAVICIFSAPNYCDTYDNRAAVVMVNR
NVMSIRQFNSSLHPYYLPNFMNAFTWSLPFVEEKLLDFGMTILHPIDGSEDHLFVDGAEAAGVATTAAPTPAANTDGSDGSVLEQRGENIREKILAMGRLSRMF
HTLCEGNENSLTPKELAGGILPQGGLPCGPSSVQAGIRGVQQSRKTDSVNGRRQDSPIESDLEPQPRATTPVLSATPSSTGSR

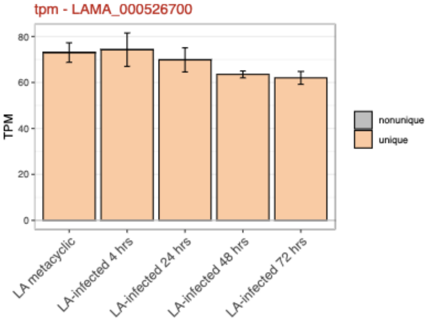

References

Fernandes et al., (2016). Dual Transcriptome Profiling of Leishmania-Infected Human Macrophages Reveals Distinct Reprogramming Signatures. *mBio*, *7*(3), e00027-16. https://doi.org/10.1128/mBio.00027-16.

Amos et al., (2022). VEuPathDB: the eukaryotic pathogen, vector and host bioinformatics resource center. *Nucleic acids research*, *50*(D1), D898–D911. https://doi.org/10.1093/nar/gkab929.

Shanmugasundram, et al., (2023). TriTrypDB: An integrated functional genomics resource for kinetoplastida. *PLoS neglected tropical diseases*, *17*(1), e0011058. https://doi.org/10.1371/journal.pntd.0011058.
