# Supplementary figures and images for: Stage-Specific Expression and Subcellular Localization of Calcineurin in Infective Forms of Leishmania amazonensis
Source: Pathogens. 2025 Nov 10;14(11):1139. doi: 10.3390/pathogens14111139 (PMC12655812; doi:10.3390/pathogens14111139)

# Figure S2

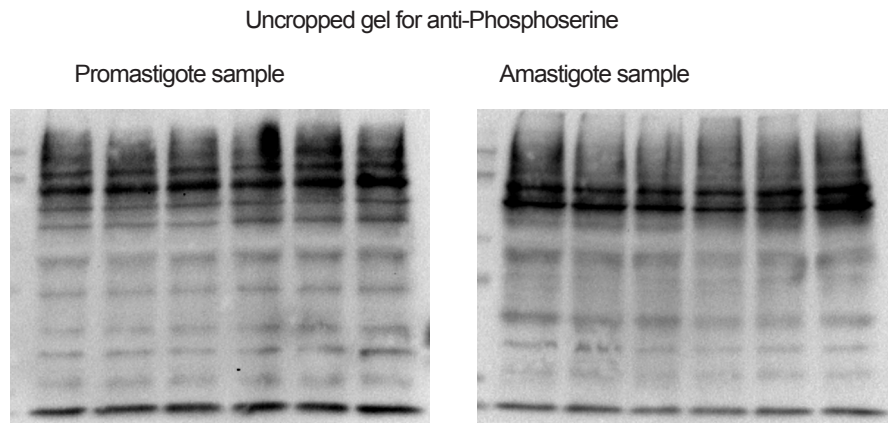

# Figure S3

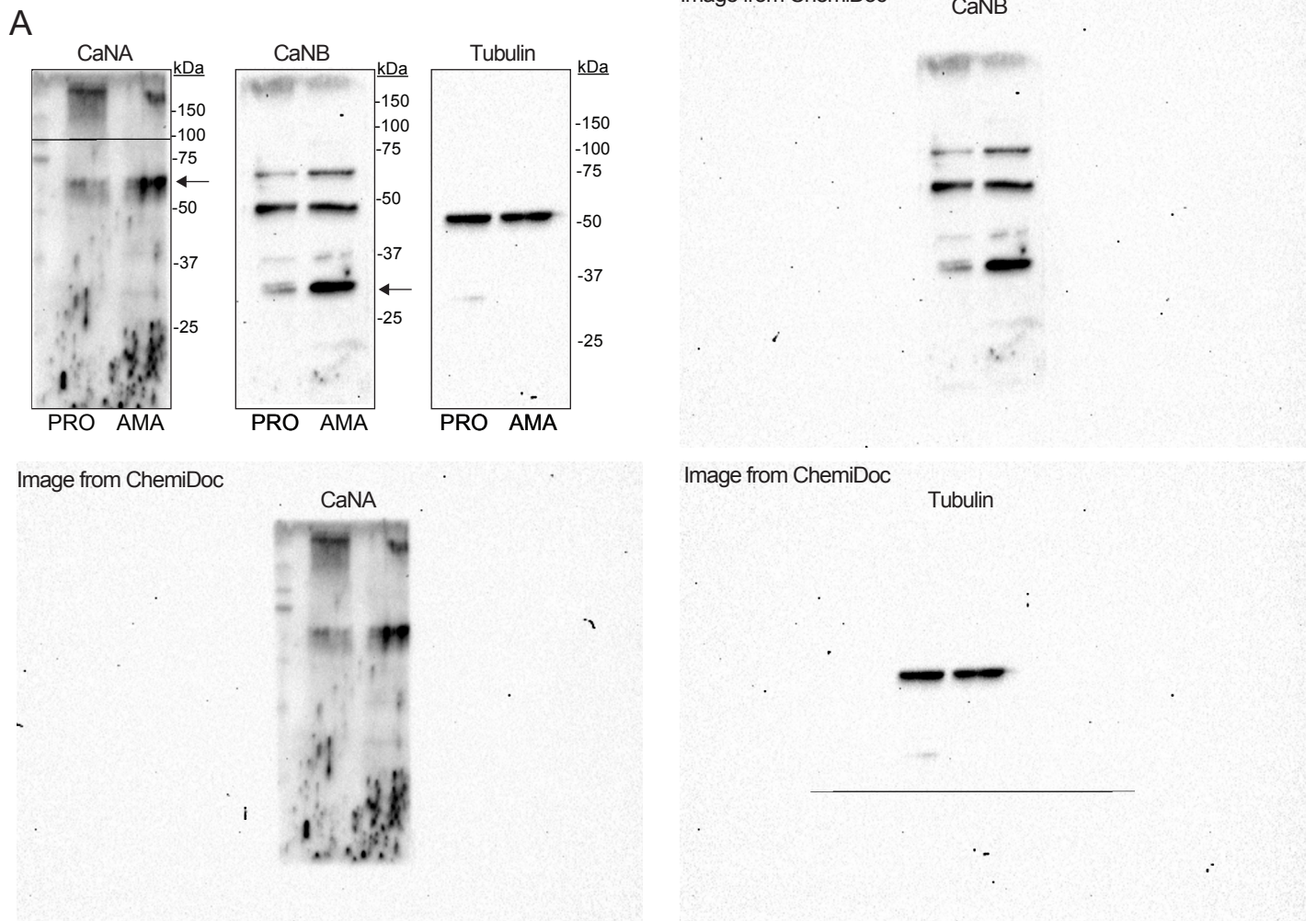

Supplement: Supplementary file 1 [file pathogens-14-01139-s001.zip › pathogens-3900273-supplementary/pathogens-3900273-File S1. Original Western blot images.pdf]
